# Predictive models for prolonged hospitalization following major hemorrhage: a retrospective study

**DOI:** 10.3389/fmed.2026.1968934

**Published:** 2026-10-29

**Authors:** Yang Bai, Jing Bai, Yu-Kun Xu, Wei-Chao Zheng, Yue Fu, Ying Sun, Hao-Yang Geng

**Affiliations:** 1 Department of Anesthesiology, Hebei Province Cangzhou Hospital of Integrated Traditional Chinese and Western Medicine, Cangzhou, China; 2 Hebei Province Key Laboratory of Integrated Traditional and Western Medicine in Neurological Rehabilitation, Cangzhou, China; 3 Department of Anesthesiology, Cangzhou Central Hospital, Cangzhou, China

**Keywords:** body mass index, fluid resuscitation, hemorrhagic shock and resuscitation, postoperative pulmonary inflammation, prolonged hospital stay, renal function

## Abstract

**Objective:**

This retrospective study aimed to identify the perioperative risk factors associated with prolonged hospital stays in patients undergoing hemostatic resuscitation for intraoperative massive bleeding.

**Methods:**

The retrospective analysis included clinical data from 119 patients who received intraoperative resuscitation for intraoperative massive hemorrhage. Patients were divided into two groups based on length of hospital stay, using the 3rd quartile as the cutoff (> 26 days vs. ≤ 26 days). The Mann–Whitney U test or independent samples t-test analyzed the influence of categorical variables on hospital stay. Chi-square test was used for count data, and continuous variables were analyzed using Spearman correlation. Risk factors prolonging hospitalization were identified and predictive models developed.

**Results:**

Multivariate analysis revealed that independent risk factors for prolonged hospitalization following major hemorrhage comprised unplanned reoperation (OR = 6.743, *P* = 0.008), admission to the intensive care unit (ICU) (OR = 9.294, *P* = 0.020), impaired renal function (OR = 11.554, *P* < 0.001), Postoperative pulmonary inflammation (OR = 4.696, *P* = 0.018) and body mass index (BMI) (OR = 10.030, *P* = 0.046). The area under the receiver operating characteristic curve (AUC) of the constructed predictive model was 0.908.

**Conclusion:**

Our study identified potentially risk factors, like BMI and abnormal kidney function, prolonging hospitalization after intraoperative massive hemorrhage. The predictive model may aid in identifying high-risk patients needing aggressive management to minimize complications. However, it is imperative to conduct prospective, high-quality research in order to validate our findings in the future.

## Introduction

Excessive intraoperative hemorrhage, a pervasive complication in surgery, engenders diminished effective circulation of blood. This consequently precipitates reduced cardiac output, jeopardizes perfusion of organs and tissues, and constrains systemic oxygen delivery (1). Expeditious and rapid fluid resuscitation, coupled with replenishment of circulating blood volume, fulfills a pivotal role in managing patients with massive hemorrhage (2–4). However, it is imperative to recognize that notwithstanding timely and efficacious resuscitation, the specter of ischemia-reperfusion injury to tissues and organs persists. Previous investigations demonstrate that resuscitation from massive hemorrhage can elicit ischemia-reperfusion injury in diverse tissues and organs (5, 6). These factors profoundly contribute to prolonged hospitalization and ominous prognosis, imposing a formidable burden on families and society.

The occurrence of postoperative complications is one of the main causes of prolonged hospitalization, including surgical site infection and postoperative pulmonary inflammation (7, 8). Diminished renal blood flow induced by intraoperative hemorrhage constitutes a cardinal factor contributing to postoperative renal dysfunction, which can potentially advance to acute kidney injury in severe cases (9). Earlier studies established that acute kidney injury is an instrumental factor contributing to extended hospitalization and heightened mortality among patients with traumatic hemorrhagic shock (10, 11). While fluid resuscitation fulfills an indispensable role in massive hemorrhage management, it is noteworty that immoderate administration of crystalloid fluids may exert adverse effects on renal function (12).

However, there is limited knowledge of the specific perioperative risk factors that contribute to prolonged hospitalization following major intraoperative bleeding. Current studies primarily focus on individual postoperative complications, such as surgical site infections or pulmonary conditions, as contributors to prolonged hospitalization. These studies lack an integrated approach that considers a combination of perioperative factors to predict extended hospital stays. To address this knowledge gap, our study develops a comprehensive predictive model that integrates these risk factors, aiming to provide clinicians with a more holistic and practical tool to predict and prevent prolonged hospitalization.

## Materials and methods

### General information

The clinical data of patients undergoing surgery at Hebei Cangzhou Hospital of Integrated Traditional Chinese and Western Medicine (Hebei Province, China) between January 1 and August 31, 2022, and experienced intraoperative blood loss exceeding 800 ml were retrospectively collected (Figure. 1). During the study period, 141 potentially eligible patients were initially identified. After 22 patients were excluded according to the predefined exclusion criteria, 119 patients were included in the final analysis. The inclusion criteria encompassed: (1) The intraoperative blood loss surpassing 800 ml, complete data availability, and survival at discharge; (2) Incorporating thoracic, abdominal, limb and pelvic (open reduction and internal fixation of pelvis), and spinal surgeries (spinal decompression). Exclusion criteria include: (1) Severe endocrine disorders, or immune system abnormalities leading to prolonged postoperative hospitalization; (2) People with existing organ failure, serious underlying diseases (heart, lung, liver, kidney dysfunction); (3) Surgery for craniocerebral injury or severe trauma requiring multiple surgical treatments; (4) Patients whose condition worsens and eventually dies and whose data is incomplete.

**Figure 1:**
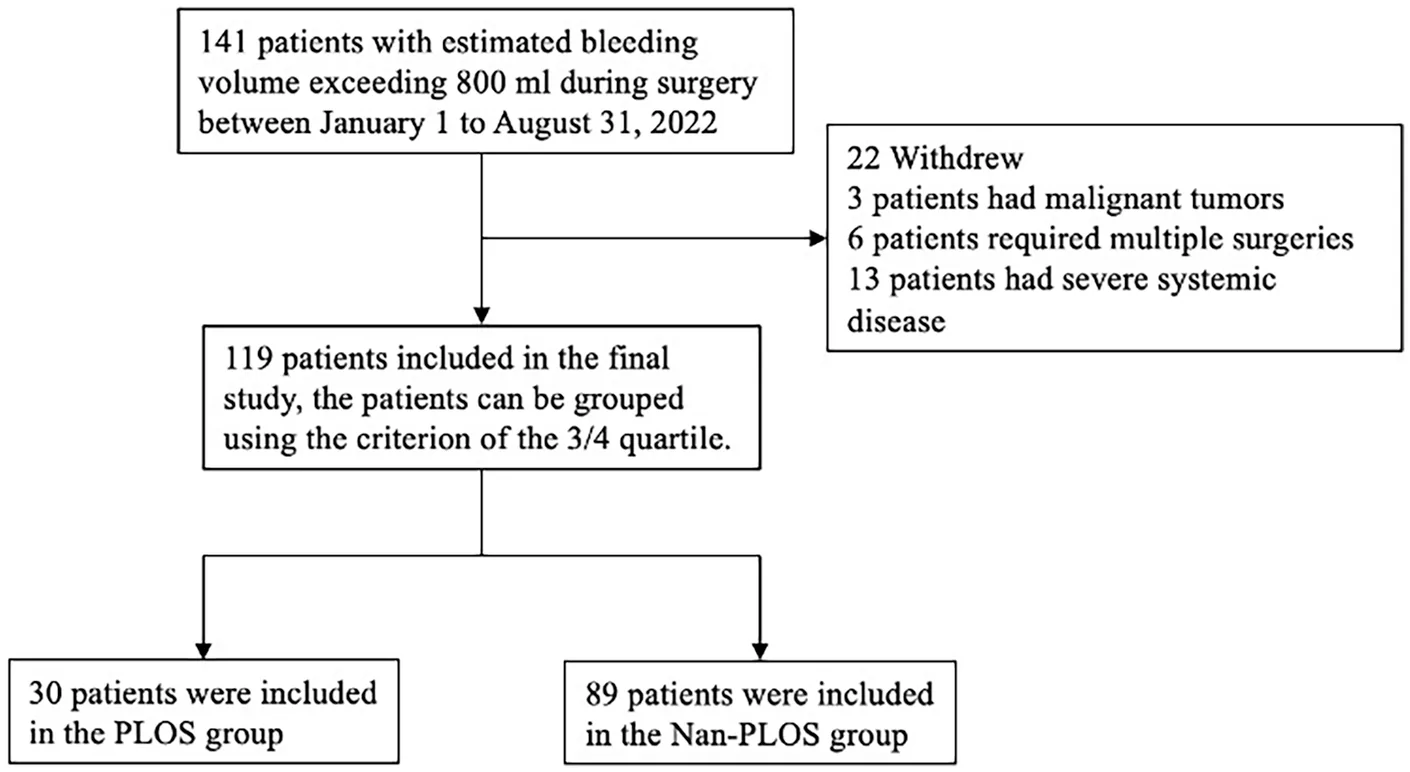
Enrollment of patients and assessment of primary outcomes. PLOS, the prolonged length of stay; No PLOS, the non-prolonged length of stay.

### Grouping

In this study, the duration of postoperative hospitalization was defined as the interval from the day following surgery until the day of discharge. A comprehensive cohort of 119 patients was enrolled for analysis. Building upon previous literature, postoperative hospitalization surpassing the 75th percentile among all patients was categorized as prolonged postoperative hospitalization. Patients were then divided into two distinct groups: the prolonged length of stay group (PLOS group; *n* = 30; with a hospitalization period exceeding 26 days), and the non-prolonged length of stay group (No PLOS group; *n* = 89; with a hospitalization period of 26 days or fewer), utilizing a criterion of a 3/4 score (13).

### Data collection

Non-emergency surgeries with intraoperative blood loss exceeding 800 ml were included, encompassing procedures such as open reduction and fixation of upper and lower limb fractures, thoracoabdominal mass resection, pelvic fracture incision, and spine surgery. Data collection for this study was performed by trained administrators who retrieved electronic medical records, Neusoft anesthesia and perioperative information system (APMIS), nursing documents, medical records department files, and other pertinent materials.

### Observation indicators

This section presents pertinent indicators that could lead to prolonged postoperative hospitalization for patients. Variables encompassed age, gender, height, weight, American Society of Anesthesiologists (ASA) Physical Status Classification System; systolic and diastolic arterial pressures, heart rate, preoperative hemoglobin, prealbumin, serum creatinine, preoperative blood urea nitrogen, coagulation parameters, surgery type; preexisting comorbidities including hypertension, coronary artery disease, type 2 diabetes mellitus, chronic obstructive pulmonary disease; intraoperative metrics including anticipated intraoperative hemorrhage volume, urine output, crystalloid infusion quantity (sodium potassium magnesium calcium glucose injection), colloid infusion (6% HES 130/0.4), red blood cell (RBC) transfusion, surgical duration, anesthesia time; postoperative indices and complications including hemoglobin, albumin, serum creatinine, blood urea nitrogen, renal dysfunction (Serum creatinine is greater than 133umol/L), pneumonitis, surgical site infection, unplanned reoperation, intensive care unit admission, with postoperative complications authenticated per clinical standards. Standardized risk factor questionnaires and scales were completed in a uniform manner and assessed utilizing unified criteria. Accrued data were documented utilizing Excel software and subsequently analyzed by domain experts.

The postoperative variables, including ICU admission, unplanned reoperation, renal dysfunction, and postoperative pulmonary inflammation, were assessed during the postoperative period, starting from the day of surgery and throughout the hospitalization. ICU admission and unplanned reoperation were captured from medical records and perioperative notes, while renal dysfunction and postoperative pulmonary inflammation were diagnosed based on clinical assessments, laboratory findings, and imaging studies documented in patient records. The inclusion of these variables in the predictive model reflects their significant potential to influence prolonged hospitalization, as identified through prior research and our analysis. Postoperative pulmonary (between 24 h and 2 weeks after surgery) inflammation is defined as the following conditions. The patient has cough, thick phlegm, wet rales in the lungs, and one of the following conditions: (1) Fever. (2) The total number of white blood cells and/or the proportion of neutrophils increases. (3) x-ray examination showed inflammatory infiltrative lesions in the lungs.

### Statistical methods

Data analysis was performed using Prism version 9.0 software. Quantitative data that followed a normal distribution were presented as mean ± SD, and independent sample t-tests were employed for intergroup comparisons. Non-normally distributed quantitative data were analyzed using non-parametric tests. Qualitative data were described statistically using frequency, composition ratios, or rates, and analyzed using the chi-square test. Based on the results of univariate analysis and in accordance with previous studies, a binary logistic regression analysis was performed using the occurrence of postoperative PLOS as the dependent variable (non-PLOS = 0, PLOS = 1), while including independent variables that exhibited statistically value less than 0.1 (*P* < 0.1). Body mass index (BMI) was analyzed as a continuous variable for descriptive statistics but was categorized into two groups (BMI ≥ 25 and BMI < 25) for regression analysis, based on clinically relevant thresholds. This dichotomization facilitated better model interpretability and clinical applicability. A binary logistic regression model was employed for multivariate analysis to identify independent risk factors associated with prolonged hospital stay and to establish a predictive model. The effectiveness of the model was evaluated by generating a Receiver Operating Characteristic (ROC) curve, plotting sensitivity (*y*-axis) against 1-specificity (*x*-axis). Internal validation of the predictive model was performed using the bootstrap resampling method. A total of 1,000 bootstrap repetitions were conducted to evaluate the stability and accuracy of the model's predictive performance. During each iteration, a random sample was drawn with replacement from the original dataset, and the model was recalibrated to assess its performance in predicting prolonged hospitalization. A significance level of *P* *<* *0.05* was considered indicative of statistical significance.

## Results

Ultimately, 22 patients were excluded from the study. Exclusions included 3 patients with malignancy who were hospitalized for long periods of time, 6 patients who required multiple surgical interventions, and 13 patients with severe preoperative systemic comorbidities who required hospitalization for other treatments in addition to surgical treatment. The study involved a total of 119 patients, 67 men and 52 women. Among them, 30 patients belonged to the PLOS group and the remaining 89 patients belonged to the non-PLOS group (Figure 1).

### Patient characteristics

The final cohort included 119 patients who were all Han Chinese, with the following characteristics: average age, 60 ± 17 years; female, 43.7%; BMI, 25.6 ± 3.6 kg/m^2^; (Table 1). The results of the univariate analysis unveiled notable discrepancies between the PLOS and non-PLOS groups in terms of height, heart rate, ASA grade, and serum creatinine. Specifically, these variables exhibited significantly higher levels in the PLOS group compared to the No PLOS group, with statistically significant differences observed (*P* *<* *0.05*). Conversely, when examining weight, age, gender, hemoglobin, prealbumin, coagulation function, entry systolic blood pressure, diastolic blood pressure, and surgical site between the two groups, no statistically significant differences were identified (*P* *>* *0.05*) (Table 1).

**Table 1:** Baseline characteristics of patients.

Characteristics and variables	All patients (*n* = 119)	PLOS group (*n* = 30)	No PLOS group (*n* = 89)	*P* value
Age, years, median (IQR)	60 (17)	57 (22)	61 (18)	0.109
Sex, female, number (%)	52 (43.7)	11 (36.7)	41 (46.1)	0.160
Weight, kg, median (IQR)	72 (17.5)	76 (24.0)	69 (14.0)	0.136
Height, cm, median (IQR)	169 (14)	171 (12)	169 (12)	0.028
BMI, kg/m^2^, mean (SD)	25.57 (3.64)	26.32 (4.13)	25.43 (3.91)	0.077
ASA, score, II (%)	95 (79.7)	8 (26.6)	87 (97.7)	0.001
SBP, mm Hg, median (IQR)	145 (31.5)	158 (181.0)	138 (29.0)	0.946
DBP, mm Hg, median (IQR)	82 (17.5)	71 (33.0)	82 (16.0)	0.278
Heart rate, beats/minute, median (IQR)	79.5 (17.8)	85.5 (21.4)	75.3 (15.6)	0.031
Hemoglobin, g/L, mean (SD)	124.8 (21.5)	119.4 (13.6)	125.8 (14.7)	0.103
Prealbumin, mg/L, mean (SD)	151.5 (60.2)	161.5 (60.2)	148.4 (54.3)	0.097
SCr, μmol/L, mean (SD)	83.65 (36.13)	94.73 (24.57)	87.56 (28.43)	0.045
BUN, mmol/L, mean (SD)	5.56 (2.43)	6.35 (1.72)	5.78 (1.84)	0.539
APTT, seconds, median (IQR)	26.80 (4.00)	27.65 (5.60)	26.70 (5.25)	0.103
FIB, g/L, median (IQR)	2.89 (1.47)	2.06 (2.09)	2.97 (1.33)	0.132
INR, score, median (IQR)	0.99 (0.11)	1.04 (0.18)	0.98 (0.09)	0.478
PT, seconds, median (IQR)	11.47 (1.7)	11.68 (2.0)	11.44 (0.14)	0.589
TT, seconds, mean (SD)	17.68 (1.47)	17.64 (1.24)	17.68 (1.50)	0.659
Abdominal operation, number (%)	19 (16.0)	6 (16.7)	13 (14.6)	0.587
Limb and pelvis operation, number (%)	57 (47.9)	17 (56.7)	40 (44.9)	0.276
Spinal operation, number (%)	35 (29.4)	5 (16.7)	30 (33.7)	0.301
Chest operation, number (%)	8 (6.7)	3 (10.0)	5 (5.6)	0.132
Hypertension, number (%)	63 (52.9)	17 (56.6)	46 (51.7)	0.087
Coronary heart disease, number (%)	27 (22.7)	8 (26.6)	19 (21.3)	0.550
Type 2 diabetes, number (%)	13 (10.9)	4 (13.3)	9 (10.1)	0.796
Old cerebral infarction, number (%)	10 (8.4)	3 (10.0)	7 (7.8)	0.868
COPD, number (%)	10 (8.4)	4 (13.3)	6 (6.7)	0.694

Categorical value is displayed as count (percentage). Continuous variables are displayed as mean (SD) and median (IQR).

PLOS, the prolonged length of stay; No PLOS, the non-prolonged length of stay, ASA, American Society of Anesthesiologists; SBP, Systolic blood pressure; DBP, Diastolic blood pressure; SCr, Serum creatinine; BUN, Blood urea nitrogen; APTT, activated partial thromboplastin time; FIB, fibrinogen; INR, international normalized ratio; PT, prothrombin time; TT, thrombin time; COPD, chronic obstructive pulmonary disease. A significance level of *P* *<* *0.05* was considered indicative of statistical significance.

### Intraoperative variables

There were no statistically significant differences observed between the two groups (*P* *>* *0.05*) in terms of preoperative comorbidities, including hypertension, coronary heart disease, type 2 diabetes, old cerebral infarction, and chronic obstructive pulmonary disease. Similarly, there were no significant variations in intraoperative estimated blood loss, urine volume, crystal infusion volume, red blood cell infusion volume, hydroxyethyl starch 130/0.4 infusion volume (6% HES 130/0.4), surgical duration, and anesthesia time (Table 2).

**Table 2:** Perioperative characteristics of patients.

Characteristics and variables	All patients (*n* = 119)	PLOS group (*n* = 30)	NO PLOS group (*n* = 89)	*P* value
Estimated blood loss, mL/kg, mean (SD)	18.73 (7.55)	18.35 (9.88)	18.88 (6.50)	0.142
Urine volume, mL/kg, mean (SD)	17.60 (9.55)	18.01 (11.47)	17.44 (8.78)	0.842
Isotonic crystalloid transfusion, mL/kg, mean (SD)	34.23 (11.37)	34.69 (11.86)	34.05 (11.25)	0.915
Hydroxyethyl starch 130/0.4, mL/kg, mean (SD)	14.62 (5.46)	15.34 (6.52)	14.33 (5.00)	0.241
RBC transfusion, mL/kg, median (IQR)	11.00 (8.99)	14.33 (14.64)	11.00 (7.89)	0.635
Surgical duration, minutes, median (IQR)	265.0 (135)	287.5 (176)	264.0 (129)	0.239
Anesthesia time, minute, median (IQR)	332 (134.5)	366 (217.0)	331 (122.0)	0.187
Hemoglobin, g/L, median (IQR)	128 (32.00)	105 (32.25)	129 (37.00)	0.048
SCr, μmol/L, mean (SD)	83.65 (36.13)	94.73 (24.57)	87.56 (28.43)	0.103
BUN, mmol/L, mean (SD)	5.56 (2.43)	6.35 (1.72)	5.78 (1.84)	0.736
Renal Dysfunction, number (%)	31 (26.0)	19 (63.3)	12 (14.6)	0.001
Albumin, mg/L, mean (SD)	39.4 (14.6)	34.4 (9.3)	40.5 (13.8)	0.014
Pulmonary inflammation, number (%)	25 (21.0)	15 (50.0)	10 (11.2)	0.001
Unplanned reoperation, number (%)	25 (21.0)	18 (60.0)	7 (7.8)	0.001
Surgical site infection, number (%)	11 (9.2)	6 (20.0)	5 (5.6)	0.038
ICU, number (%)	13 (10.9)	8 (26.7)	5 (5.6)	0.004

Categorical value is displayed as count (percentage). Continuous variables are displayed as mean (SD) and median (IQR).

PLOS, the prolonged length of stay; No PLOS, the non-prolonged length of stay; SCr, Serum creatinine; BUN, Blood urea nitrogen; ICU, admission to the intensive care unit. A significance level of *P* *<* *0.05* was considered indicative of statistical significance.

There were significant differences in postoperative indexes observed between the two groups. The findings from the univariate analysis revealed that the PLOS group exhibited markedly lower levels of hemoglobin and albumin in comparison to the No PLOS group, with statistical significance (*P* *<* *0.05*). Furthermore, the incidence of serum creatinine, urea nitrogen, abnormal kidney function, postoperative pulmonary inflammation, proportion of unplanned reoperation, surgical site infection, and postoperative transfer to the ICU were notably higher in the PLOS group, demonstrating statistical significance (*P* *<* *0.05*) when compared to the No PLOS group (Table 2).

### Multi-variate logistic regression analysis

Based on the findings of the univariate analysis, a binary logistic regression analysis was performed using the occurrence of postoperative PLOS as the dependent variable (non-PLOS = 0, PLOS = 1), while including independent variables that exhibited statistically value less than 0.1 (*P* < 0.1). The results indicated that unplanned reoperation (OR = 6.743, *P* = 0.008, 95% CI: 1.702-29.307), ICU (OR = 9.294, *P* = 0.020, 95% CI: 1.489-69.032), impaired renal function (OR = 11.554, *P* < 0.001, 95% CI: 3.328-46.222), postoperative pulmonary inflammation (OR = 4.696, *P* = 0.018, 95% CI: 1.313-17.65) and body mass index (BMI) (OR = 10.030, *P* = 0.046, 95% CI: 1.317-127.467) were identified as independent risk factors for PLOS following resuscitation of massive hemorrhage (Table 3; Figure 2).

**Table 3:** Multivariate logistic regression model: associations between candidate variables and prolonged postoperative hospital stay.

Variable	Odds ratio	95% confidence interval	*P*-value
Body mass index	10.03	1.317–127.47	0.046
Pulmonary inflammation	4.696	1.313–17.667	0.018
Impaired renal function	11.554	3.328–46.222	0.000
Admission to the intensive care unit	9.294	1.489–69.032	0.020
Unplanned reoperation	6.743	1.702–29.307	0.008
ASA Physical Status Classification System	0.776	0.14–3.876	0.761
Post-operative infection	0.454	0.071–2.474	0.379

A significance level of *P* *<* *0.05* was considered indicative of statistical significance.

**Figure 2:**
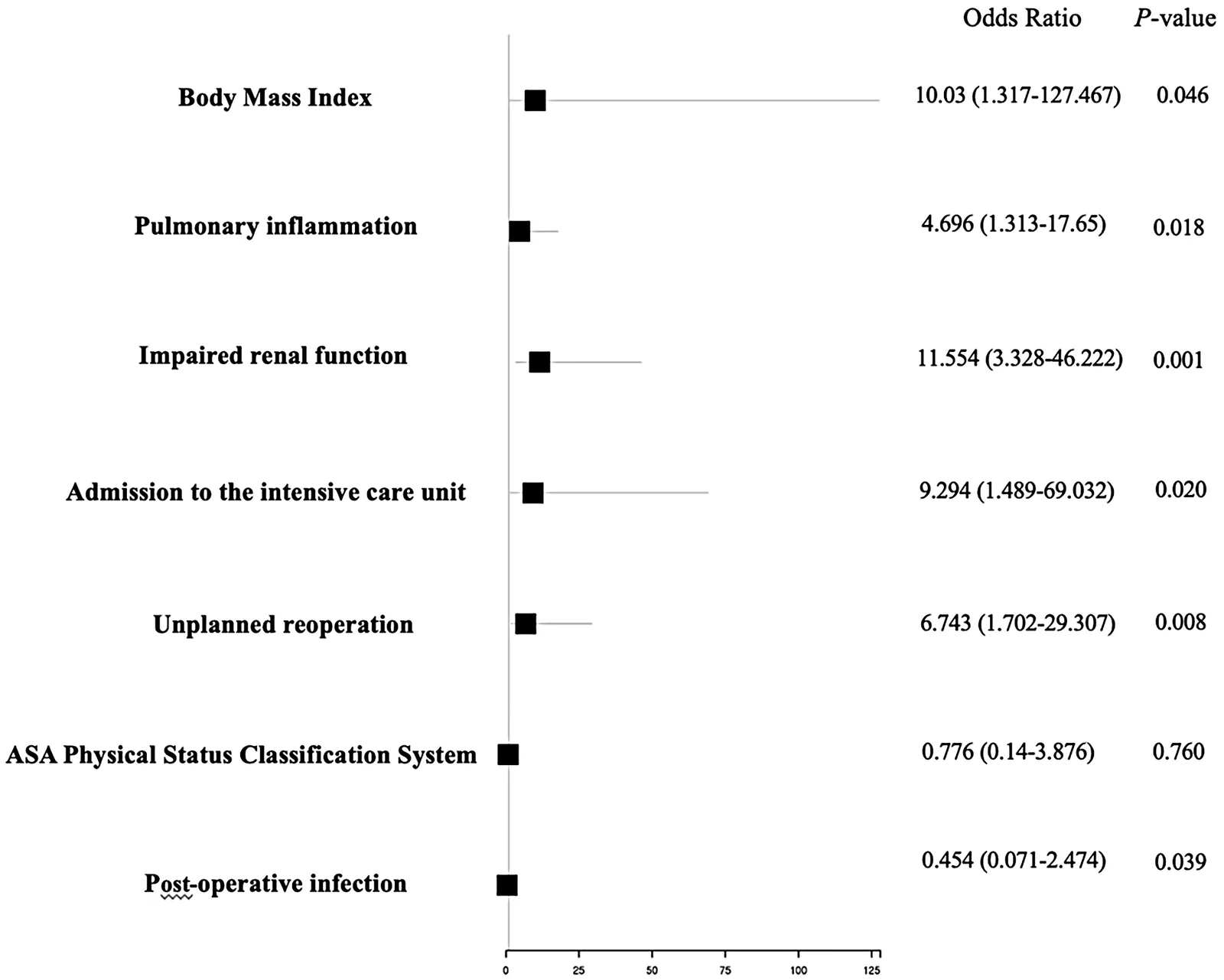
Forest plot for multiple regression.

### Model performance

The predictive model was constructed based on the logistic regression analysis results, incorporating variables such as ICU, unplanned reoperation, and BMI: Logit(P) = −4.784 + 2.229 * ICU + 1.908 * unplanned reoperation + 2.306 * BMI + 2.447 * Abnormal kidney function + 1.547 * Pulmonary inflammation. Furthermore, a visually appealing nomogram and an ROC curve were generated, with the Area Under the Curve (AUC) measuring 0.908 (Figures 3, 4).

**Figure 3:**
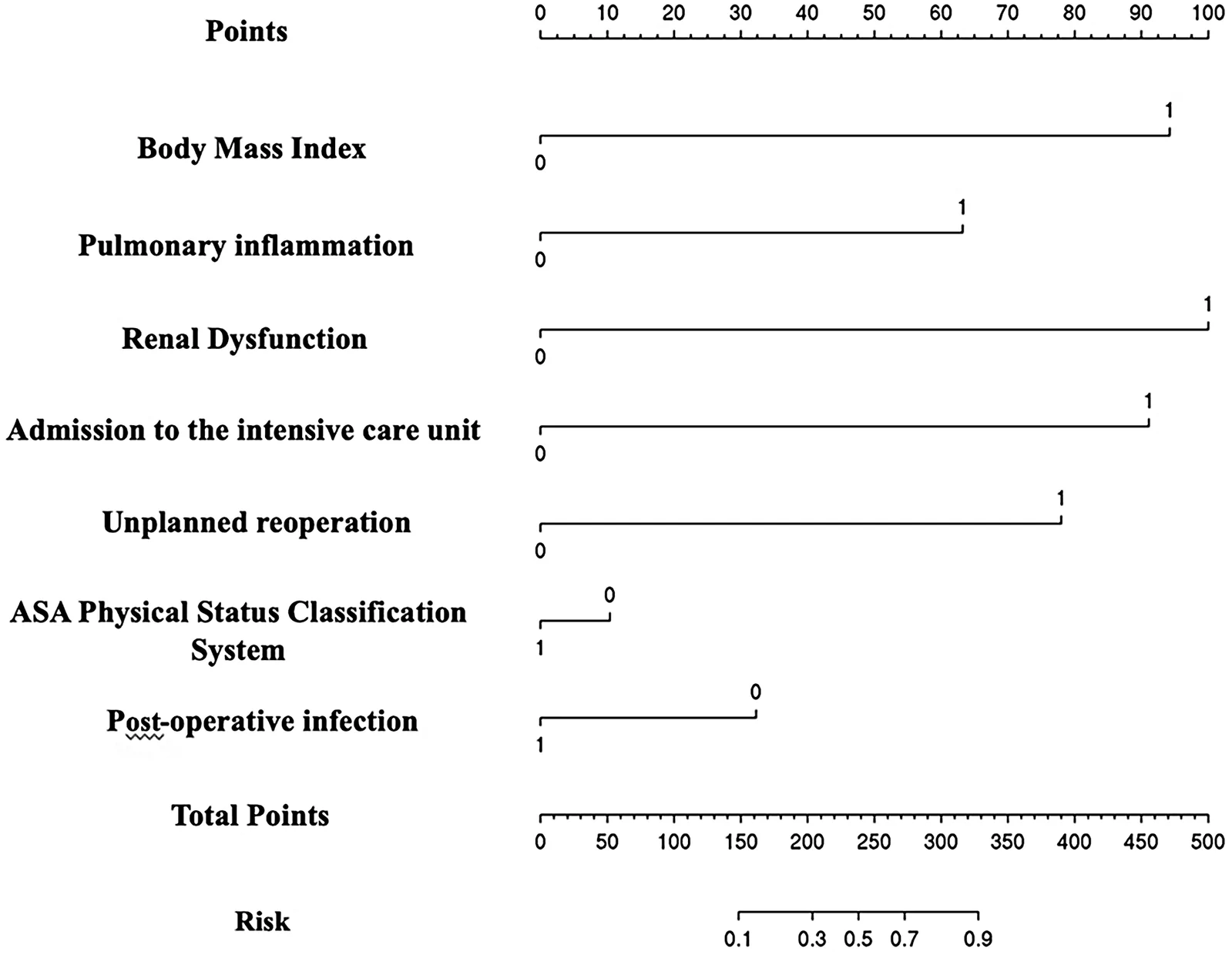
Nomogram of a multivariable prediction model for prolonged hospitalization after major hemorrhage.

**Figure 4:**
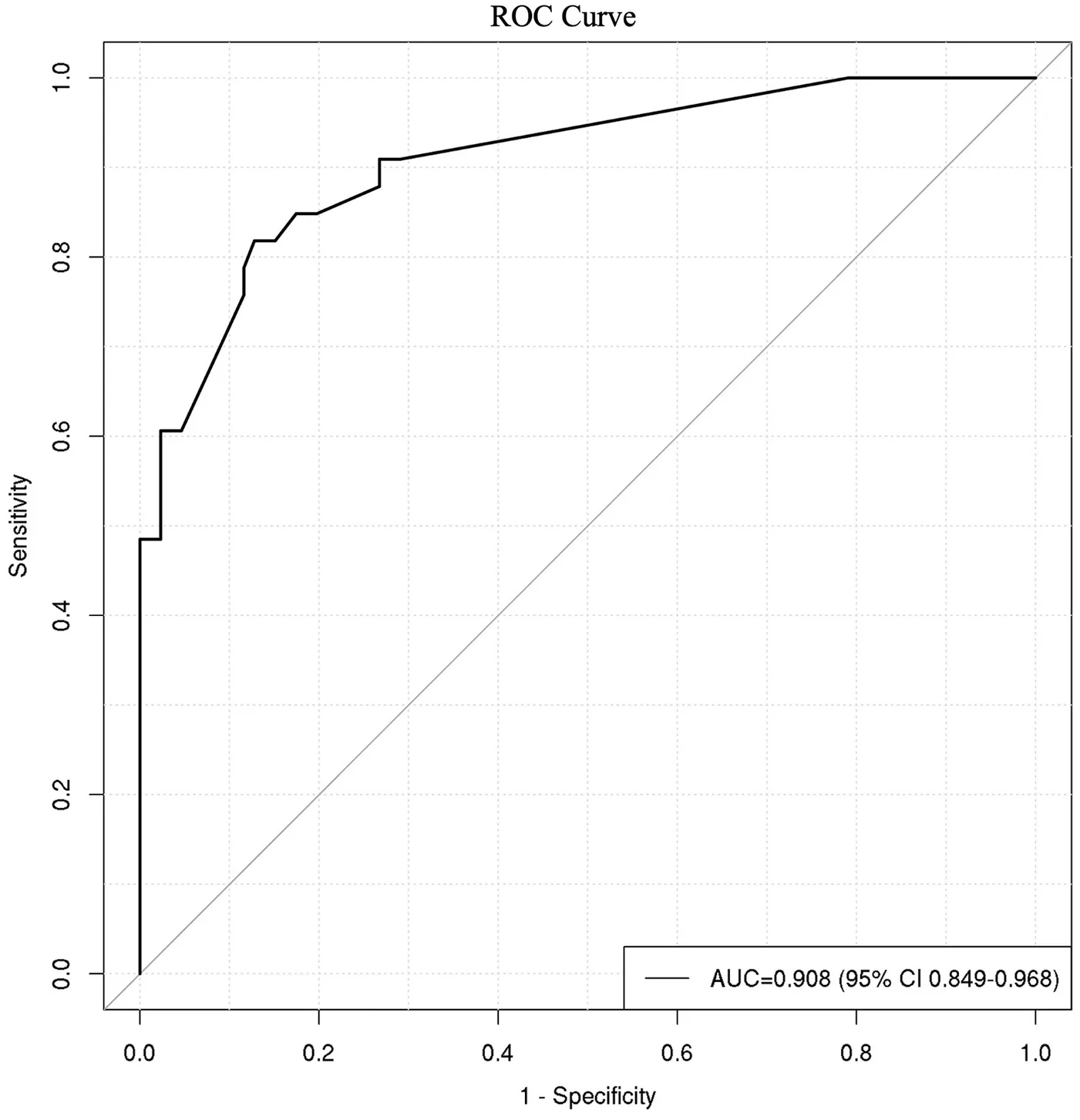
Receiver operating characteristic curve of the prediction model in the development cohort.

The Hosmer-Lemeshow goodness of fit test was performed, revealing a Brier Score of 0.107 in the calibration curve. These findings indicate that the model exhibited excellent fit and demonstrated a strong agreement between the predicted risk of prolonged hospital stay in HSR patients and the observed risk. The calibration curve was generated using a bootstrap resampling method with 1,000 repetitions, and the bias-corrected calibration curve, shown in Figure 5, illustrates the strong agreement between predicted and observed probabilities. (Figure 5).

**Figure 5:**
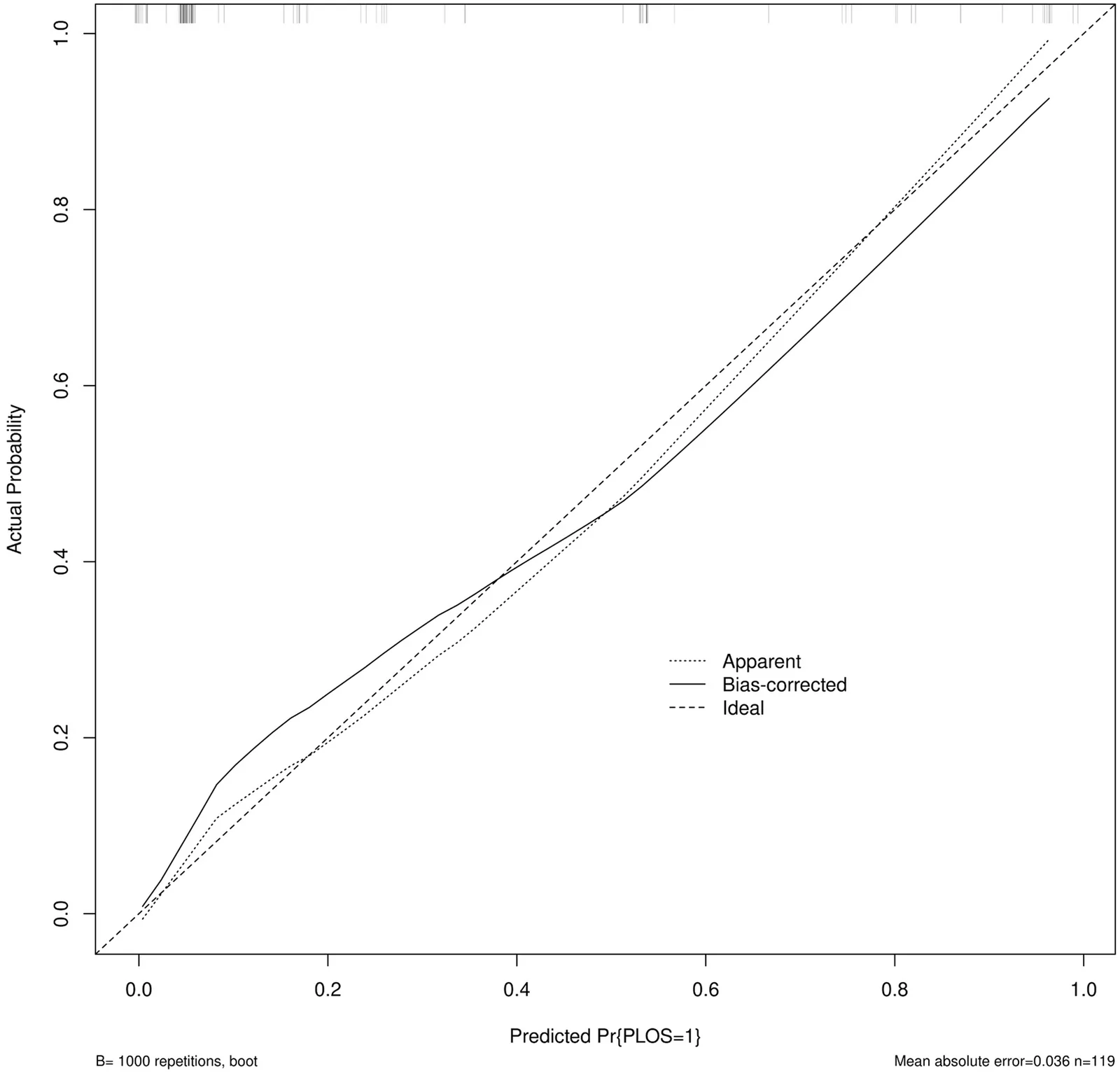
The calibration curve analysis of the nomogram.

## Discussion

The length of stay (LOS) serves as an indirect measure of surgical quality and a surrogate indicator of costs (14). This study analyzed data from 119 patients to identify risk factors contributing to prolonged hospitalization following resuscitation for major hemorrhage. Univariate analysis revealed several significant factors, including body mass index (BMI), fluid volume, and postoperative pulmonary inflammation. However, multivariable regression analysis confirmed that BMI, postoperative pulmonary inflammation, ICU admission, unplanned reoperation, and renal dysfunction were independent predictors of prolonged hospitalization. The predictive model, incorporating these variables, demonstrated strong discrimination with an area under the receiver operating characteristic curve (AUC) of 0.908 (95% CI: 0.849–0.968) and satisfactory calibration (Figures 4, 5). These findings indicate that the predicted probabilities generated by our model are closely correlated with the outcomes observed in the study cohort.

Obesity exhibits a robust correlation with an increased occurrence of postoperative complications and prolonged hospitalization (15–17). Preceding studies have illuminated that patient with excessive adiposity, as determined by overweight or obesity per body mass index (BMI), harbor an elevated susceptibility to developing major postoperative complications relative to normal weight patients (8). Furthermore, their hospitalization duration was notably extended. Our own study corroborated these findings, as we observed a higher incidence of postoperative infection among patients with elevated BMI.

Obese individuals often present with multiple underlying conditions, such as diabetes and high blood pressure, which can complicate their surgical procedures (18). Additionally, their abundant adipose tissue necessitates more intricate surgical techniques, thereby increasing the probability of surgical side injuries (19). Consequently, the risk of postoperative infection is significantly elevated in comparison to normal-weight patients. Furthermore, obese patients who have undergone resuscitation from massive hemorrhage may exhibit elevated levels of systemic inflammation, rendering them more susceptible to infections like pneumonia (20). Simultaneously, the excess adipose tissue in obese individuals releases pro-inflammatory cytokines, potentially exacerbating chronic inflammatory states (21). This chronic inflammation can impede the immune system's effectiveness in combating infections, resulting in a higher risk of postoperative infection (22).

Physiological disparities between normal-weight and obese patients should be meticulously taken into account during surgery, particularly in terms of fluid management and drug dosing to ensure optimal tissue concentration (23). By recognizing these differences, healthcare professionals can implement appropriate strategies to mitigate the risk of postoperative infections.

The decreased perfusion of multiple tissues and organs, along with the subsequent complications resulting from massive hemorrhage, are significant contributors to its high mortality rate (5, 6, 24). Severe hemorrhage triggers sympathetic nerve excitation, leading to a pronounced reduction in renal blood perfusion (25). This, in turn, causes renal ischemia and hypoxia, ultimately resulting in acute kidney injury (26). Previous studies have established that acute kidney injury is a crucial factor contributing to prolonged hospital stays and increased mortality among patients with traumatic hemorrhagic shock (27). The development of hypovolemia-associated acute kidney injury stems from the interplay between acute shock and subsequent inflammatory and coagulation disturbances (28, 29).

While crystalloid administration remains a vital component of fluid resuscitation in the management of massive hemorrhage, our study did not find a significant difference in crystalloid volume between the PLOS and non-PLOS groups. Moreover, crystalloid volume was not identified as an independent predictor in the final model. Nonetheless, it is important to note that excessive crystalloid administration has been associated with adverse effects on renal function (30–32). Our study revealed a higher incidence of abnormal kidney function among patients who received high-dose crystalloid infusions. Existing research has highlighted that as the disease progresses and anti-shock therapy takes effect, patients typically experience hemodilution and thrombocytopenia. Excessive administration of crystalloid fluids can raise hydrostatic pressure within the renal tubules, compromising the kidneys’ ability to maintain fluid balance and effectively eliminate excess fluid (33). Consequently, this exacerbates renal dysfunction. Furthermore, the duration of resuscitation and the timing of crystalloid infusion can also impact renal outcomes. Prolonged resuscitation and delayed initiation of crystalloid dosing can worsen renal ischemic injury, further heightening the risk of renal dysfunction (34).

While ICU admission, unplanned reoperation, pulmonary complications, and renal dysfunction were identified as independent risk factors for prolonged hospitalization, it is important to consider the timing of these complications during the hospital course. These complications may not only contribute to prolonged hospitalization but may also arise as part of the same clinical processes that lead to extended stays. For example, renal dysfunction and delayed recovery may result from prolonged resuscitation or excessive crystalloid infusion, which, in turn, exacerbate systemic inflammation and impair recovery. Similarly, the development of pulmonary complications, particularly postoperative pulmonary inflammation, may be secondary to prolonged immobility or impaired immune responses, further complicating recovery trajectories.

This bidirectional relationship underscores the complexity of managing critically ill patients with major hemorrhage. Identifying modifiable risk factors, such as fluid resuscitation strategies or early mobilization protocols, may help interrupt this cycle, reducing the incidence of complications and shortening hospitalization durations.

In summary, this study identified several independent risk factors for prolonged hospitalization following resuscitation for major intraoperative hemorrhage, including BMI, postoperative pulmonary inflammation, ICU admission, unplanned reoperation, and renal dysfunction. The predictive model incorporating these factors demonstrated high accuracy and satisfactory calibration, supporting its potential clinical utility. However, the generalizability of our findings may be limited by the single-center design of the study and the inclusion of a heterogeneous surgical population. While this design allowed us to identify risk factors across various surgical scenarios, the variability in patient characteristics and surgical interventions may reduce the applicability of the results to specific clinical contexts. Future multicenter studies with larger, more homogeneous cohorts are needed to validate and extend our findings, ensuring their relevance to diverse clinical settings.

### Limitation

This retrospective study aimed to examine independent risk factors associated with intraoperative major hemorrhage and prolonged hospitalization. The findings of this study have salient implications for clinical decision-making. However, the inherent limitations of this retrospective design must be acknowledged. First, the small sample size (*n* = 119), including only 30 prolonged hospitalization events, may limit the generalizability of our findings and increase the risk of statistical overfitting in the predictive model, despite the use of internal validation. Second, the definition of prolonged hospitalization was based on the cohort's 75th percentile, which may not align with universally accepted clinical thresholds, potentially limiting the comparability of our findings across different settings. Third, sensitivity, specificity, and the Youden Index were not included in this study due to the small sample size and limited outcome events, which may reduce the robustness of these metrics. We instead focused on AUC and calibration metrics, which provide a comprehensive evaluation of model performance. Finally, this study did not include perioperative coagulation assessments, such as thromboelastography (TEG), which are commonly used in major surgeries to guide transfusion strategies. Future multicenter studies with larger cohorts and standardized definitions are needed to validate and expand upon our findings.

## Conclusion

In conclusion, this retrospective study identified unplanned reoperation, intensive care unit admission, renal dysfunction, and elevated body mass index as independent risk factors for prolonged hospitalization following resuscitation for intraoperative major hemorrhage. An exploratory, internally validated prediction model was developed to assess these risks. Recognizing potentially modifiable factors, such as elevated BMI and renal impairment, offers opportunities to optimize management and improve outcomes in this critically ill patient population. Implementing preventative strategies, including prudent fluid administration to reduce the risk of complications like acute kidney injury, may contribute to shortening hospitalization duration.

## Data Availability

The original contributions presented in the study are included in the article/Supplementary Material, further inquiries can be directed to the corresponding authors.
